# Screening for sarcopenia and frailty in patients with chronic ulcers: a cross-sectional study

**DOI:** 10.1590/1677-5449.190054

**Published:** 2020-07-31

**Authors:** Tamiris Gomes, Kelly Cristina Blaszkowski Trombini, Marcos Vinicíus Soares Martins, Hilana Rickli Fiuza Martins

**Affiliations:** 1 Faculdade Guairacá, Guarapuava, PR, Brasil.; 2 Universidade Estadual do Centro-Oeste – UNICENTRO, Guarapuava, PR, Brasil.

**Keywords:** varicose ulcer, sarcopenia, frailty

## Abstract

**Background:**

Patients with venous ulcers report multiple comorbidities and are more likely to be physically inactive. Sarcopenia and frailty increase vulnerability to dependence and/or death.

**Objectives:**

To investigate the occurrence of sarcopenia and frailty in patients with chronic venous ulcers.

**Methods:**

Observational study with cross-sectional design. Nine patients (67.4 ± 8.42 years) with lower limb venous ulcers classified as CEAP 6 according to International Consensus on Chronic Venous Diseases criteria (open and active ulcer) were evaluated. Sarcopenia was assessed and classified by assessment of strength (manual dynamometry), gait speed (10-meter walk test), and muscle mass (calf circumference). Frailty screening was based on the Fried criteria, consisting of five components: unintentional weight loss; exhaustion; weakness; slow gait speed; and low physical activity.

**Results:**

Frailty was more frequent (n=9; 100%) than sarcopenia (n=1; 11,1%). The most common Fried criterion was exhaustion (n=9; 100%), followed by low physical activity (n=8; 88,8%), muscle weakness (n=5; 55%), and unintentional weight loss. Finally, the least frequent criterion was slow walking speed (n=2; 22.2%). In the subject diagnosed with sarcopenia, both weakness and reduced muscle mass were observed (n=1; 11,1%).

**Conclusions:**

Patients with chronic venous ulcers exhibit frailty or pre-frailty and the components that comprise the condition of frailty in this population are exhaustion, low physical activity, and muscle weakness. Sarcopenia was identified in a small proportion of the patients.

## INTRODUCTION

Chronic ulcers of venous origin are the most common type, accounting for as much as 80% of ulcers involving the lower limbs, and creating a serious public health problem because of the large number of people affected.^1^^,^^2^ Lower limb venous ulcers affect 1-3% of the population over the age of 60 and incidence increases with age. In turn, elderly patients with venous ulcers have multiple comorbidities and are more likely to be physically inactive.^3^

Development of chronic ulcers is multifactorial and dependent on both intrinsic and extrinsic factors. Intrinsic factors that can have an important effect on ulcer healing include changes caused by the aging process, such as changes to body composition, energy imbalances, homeostatic imbalances, and neurodegeneration.^4^

Skeletal muscle can be considered the principal component of the body’s protein content and is capable of stimulating production of antibodies, wound healing, and production of white blood cells during acute or chronic diseases. As aging reduces muscle mass, a process known as sarcopenia, there is less protein available to maintain functionality and physiological functions.^5^ The combination of reduced muscle mass and strength increases the risk of falls, hospitalizations, dependence and institutionalizations, worsening quality of life and increasing mortality, and it also has social and economic repercussions.^6^

Sarcopenia is associated with risk of frailty, risk of falls, reduced mobility, poor glycemic and metabolic control, reduced baseline metabolic rate, and lower functional capacity.^7^^,^^8^ Sarcopenia increases the risk of fractures, interferes with the capacity to perform daily activities of life and is associated with cardiac disease, respiratory disease, and cognitive impairment.^9^ Investigation of the relationship between sarcopenia and surgical morbidity in general surgery patients suggests that it is an important factor in healing of wounds and in complications.^10^

Frailty is a clinical state of weakness and susceptibility to physiological stress caused by low physiological reserves in neuromuscular, metabolic, and immunological systems.^11^ It is a clinical syndrome with multiple causes and is characterized by reduced muscle strength and reduced physical resistance and physiological function, which increases a person’s vulnerability to development of major dependence and/or likelihood of death.^12^ Elements of frailty include reduced mobility, difficulties walking, muscle weakness, reduced exercise tolerance, unstable equilibrium, poor nutrition, and sarcopenia.^13^

Since sarcopenia and frailty are strongly associated with adverse effects on health and interfere with wound healing and because chronic wounds are associated with age, comorbidities, and physical inactivity, meaning that chronic wound patients are a population with a propensity for sarcopenia and frailty, it is necessary to conduct an investigation into the prevalence of these conditions in this population, considering that, to our knowledge, no previous studies have evaluated the relationship between sarcopenia and frailty among patients with venous ulcers. Therefore, the objective of this study is to investigate the occurrence of sarcopenia and frailty in patients with chronic venous ulcers.

## METHODS

This observational, cross-sectional study was conducted with the objective of evaluating the frequency of sarcopenia and frailty in patients with chronic lower limb venous ulcers. The study was approved by the Ethics Committee at the Universidade Estadual do Centro-Oeste (UNICENTRO), Guarapuava, PR, Brazil, under ruling number 2.810.567-2018 and was conducted at the Clínicas Integradas da Faculdade Guairacá, Guarapuava, PR, Brazil. Sampling was non-probabilistic and sample recruitment was by convenience, inviting patients to take part verbally at the chronic wounds clinic run by the same institution at which the study was conducted and by distribution of pamphlets at health centers, clinics, and other health services. Nineteen patients were contacted or contacted the research team, but only 9 patients were enrolled. The material was distributed during August 2018, after approval by the Research Ethics Committee, and sample recruitment and data collection took place simultaneously, during September, October, and November of 2018.

In order to be defined as elderly, a person must have a chronological age ≥ 65 years in developed countries, or be from 50 to 64 years old and have clinical conditions or physical limitations affecting the ability to walk or to perform activities of daily living, because of their physical fitness or the physiological conditions affected.^14^ Therefore, the study enrolled patients over the age of 50 who had chronic venous ulcers classified as CEAP 6 according to the international consensus on chronic venous diseases (open and active ulcers)^15^ and who had been diagnosed with the condition by a physician. One patient who had not been diagnosed was enrolled on the study after consultation with a vascular surgeon who confirmed the condition.

Exclusion criteria were age younger than 50 years, lower-limb ulcers of arterial origin, burns, diabetes, and pressure ulcers. Patients were not enrolled on the study if they had consumed substances that could interfere with walking or if they had metabolic or endocrine diseases affecting the musculoskeletal system. Other factors leading to exclusion were unilateral or bilateral hip replacement, cardiac and/or respiratory abnormalities, self-report of acute painful conditions affecting upper or lower limbs, upper and/or lower limb amputations, stroke, Parkinson’s, cancer-related cachexia, chronic kidney disease, Alzheimer’s or psychiatric disease, severe arthritis or inflammatory disease, drug-related anorexia, lack of a means of transport to travel to examinations, and refusal to take part.

Sarcopenia was diagnosed using the algorithm proposed by the European Working Group on Sarcopenia in Older People (EWGSOP)^16^ and frailty was diagnosed using the Fried frailty phenotype.^17^ Screening for sarcopenia was based on values for gait speed (GS), hand grip strength (HGS), and calf circumference (CC), as proposed by the EWGSOP. Sarcopenia was defined as present when the patient had reduced skeletal muscle mass combined with reduced strength and/or physical performance. Sarcopenia was staged as follows: pre-sarcopenia (reduced muscle mass), sarcopenia (reduced skeletal muscle mass combined with reduced strength and/or physical performance), or severe sarcopenia (reduced skeletal muscle mass, muscle strength, and physical performance).

Frailty syndrome was identified using the five criteria proposed by Fried et al.^17^: unintentional weight loss; exhaustion assessed by self-report of fatigue; reduced HGS; low physical activity level; and reduced gait speed. Patients were classified as frail when three or more criteria were present, pre-frail when one or two were present, and not frail when none of the criteria were present.

Grip strength (kg) was tested using a digital dynamometer (Camry, EH101 model, China). Participants were seated comfortably, with elbows flexed at a 90° angle, against the trunk. They performed three attempts with a 1-minute rest between them and the mean of the three results was used for analysis. Reduction in muscle strength was defined according to sex and body mass index (BMI = body mass [kg]/height^2^ [m]). The cutoff points used for women were: ≤ 17 kg for BMI ≤ 23kg/m^2^; ≤ 17.3 kg for BMI 23.1-26 kg/m^2^; ≤ 18 kg for BMI 26.1-29 kg/m^2^; and ≤ 21 kg for BMI > 29. The cutoff points for men were ≤ 29 kg for BMI ≤ 24 kg/m^2^; ≤ 30 kg for BMI 24.1-26 kg/m^2^; ≤ 30 kg for BMI 26.1-28 kg/m^2^; and ≤ 32 kg for BMI > 28 kg/m^2^.^16^

Gait speed was assessed by the 10-meter walk test, in which participants walk a distance of 10 meters in a straight line. The first two meters and the last two meters were ignored, to allow for acceleration and deceleration and the time taken to walk the remaining six meters was recorded. This distance was divided by the time the participant took to walk the distance to give an average velocity in m/s. The test was performed three times and values of ≤ 0.8 m/s were defined as indicative of slow GS.^6^^,^^16^

Calf circumference was measured using an inelastic tape measure around the largest curvature of the calf with the participant seated on a chair with knees and hips at 90°. Measurements lower than 31 cm were defined as indicative of muscle mass depletion.^18^

Unintentional weight loss was assessed by asking the participants if they had lost 4.5 kg or more or at least 5% of their body weight in the preceding year and a positive reply was considered a criterion of frailty. Body mass was measured using a digital balance (Filizola, Brazil) accurate to 0.1 kg and height was measured using a stadiometer (Cardiomed, Brazil), and then BMI was calculated from the results.

Exhaustion/fatigue was assessed using two questions from the Center for Epidemiologic Studies-Depression scale (CES-D). Participants were asked “Have you felt that you had to make an effort to manage your everyday activities?” and “Have you felt unable to get things done?”. Responses were given on a Likert scale (never or rarely = 1, sometimes = 2, much of the time = 3, always = 4). If the patient replied much of the time and/or always for one of the two questions, fatigue was defined as present as a criterion of frailty.

Physical activity levels were assessed using the Profile of Human Activity (PHA), which is a questionnaire that has been adapted and validated for the elderly population of Brazil. The questionnaire comprises 94 items ranging from routine activities of a low functional level (sit down and get up from a chair or bed) to activities of a higher functional level (run 4.8 kilometers in less than 30 minutes). The activities are based on the energy expended: those scored lower require less energy expenditure and those scored higher require greater energy expenditure. An elderly participant is considered active if they have an adjusted activities score (AAS) > 74; moderately active if 53 > AAS < 74, and inactive if AAS< 53.^19^

Reduced gait speed was assessed according to the timed taken to walk 4.6 meters at a normal pace. Men were scored 1 point if their height was ≤ 173 cm and they took ≥ 7 seconds, or their height was ≥ 173 cm and they took ≥ 6 seconds to walk 4.6 meters. Women were scored 1 point if their height was ≤ 159 cm and they took ≥ 7 seconds or their height was ≥ 159 cm and they took ≥ 6 seconds to cover the distance of 4.6 meters.^17^

Descriptive statistics were used for analysis of the results. The descriptive analysis of the characteristics of the population consisted of calculation of absolute and relative frequencies and means and standard deviations. The statistical analysis was performed using SPSS version 23.

## RESULTS

**Figure 1:**
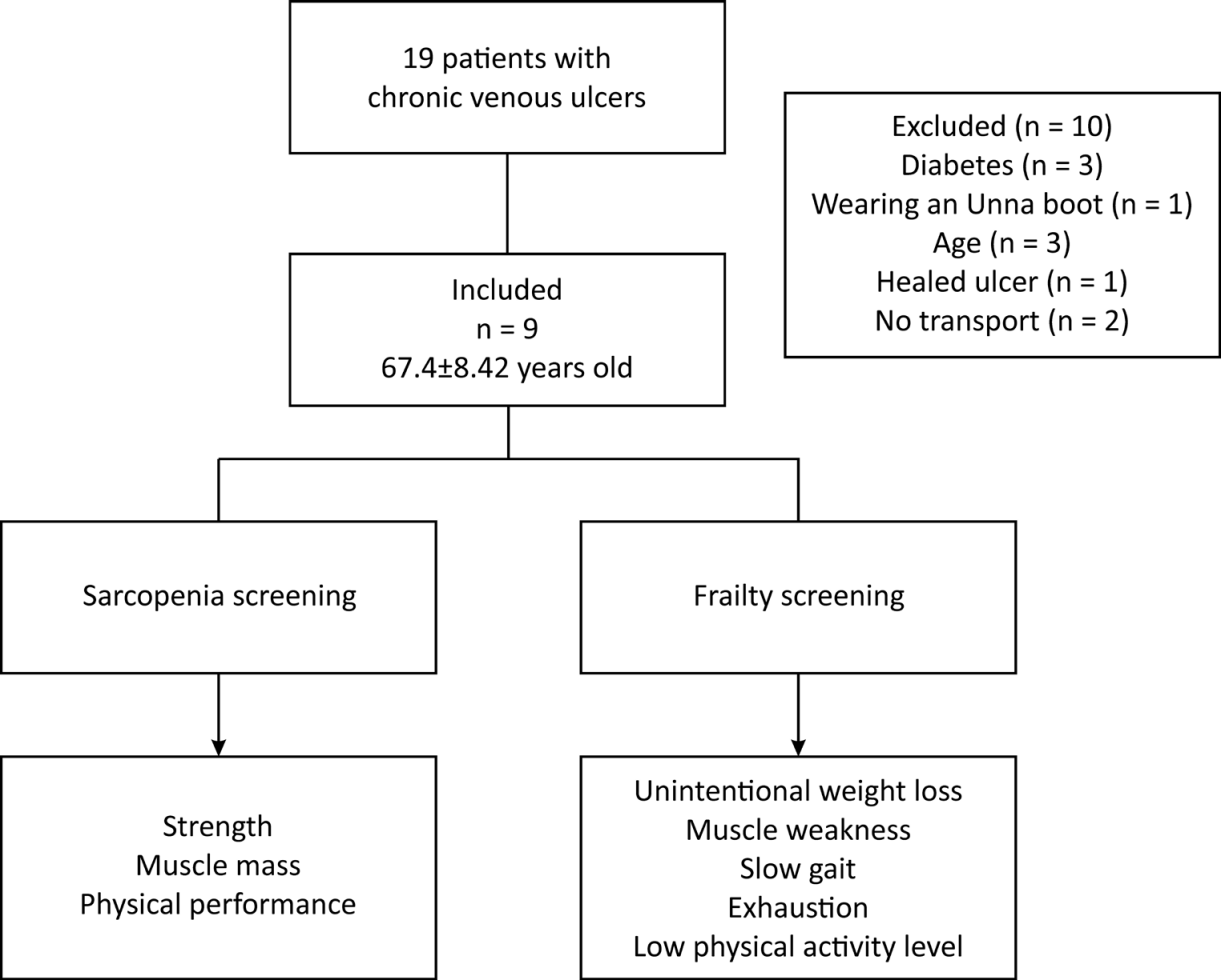
Study flow diagram.

Nine patients with venous ulcer on the lower limbs took part in the study, 66.7% (n = 6) of whom were female and 33.3% (n = 3) of whom were male, with a mean age of 67.44±8.42 years, mean body mass of 71.2±28.8 kg, and mean height of 1.63±0.11 meters (Figure 1). Six of the nine participants (66.7%) had unilateral ulcers and 33.3% (n = 3) had bilateral ulcers. The mean time since onset was 117.8±160.33 months and the mean number of active ulcers was 2.77±3.27. With relation to comorbid diseases, 66% (n = 6) of the patients had arterial hypertension and 22.2% (n = 2) had cardiovascular disease.

**Figure 2:**
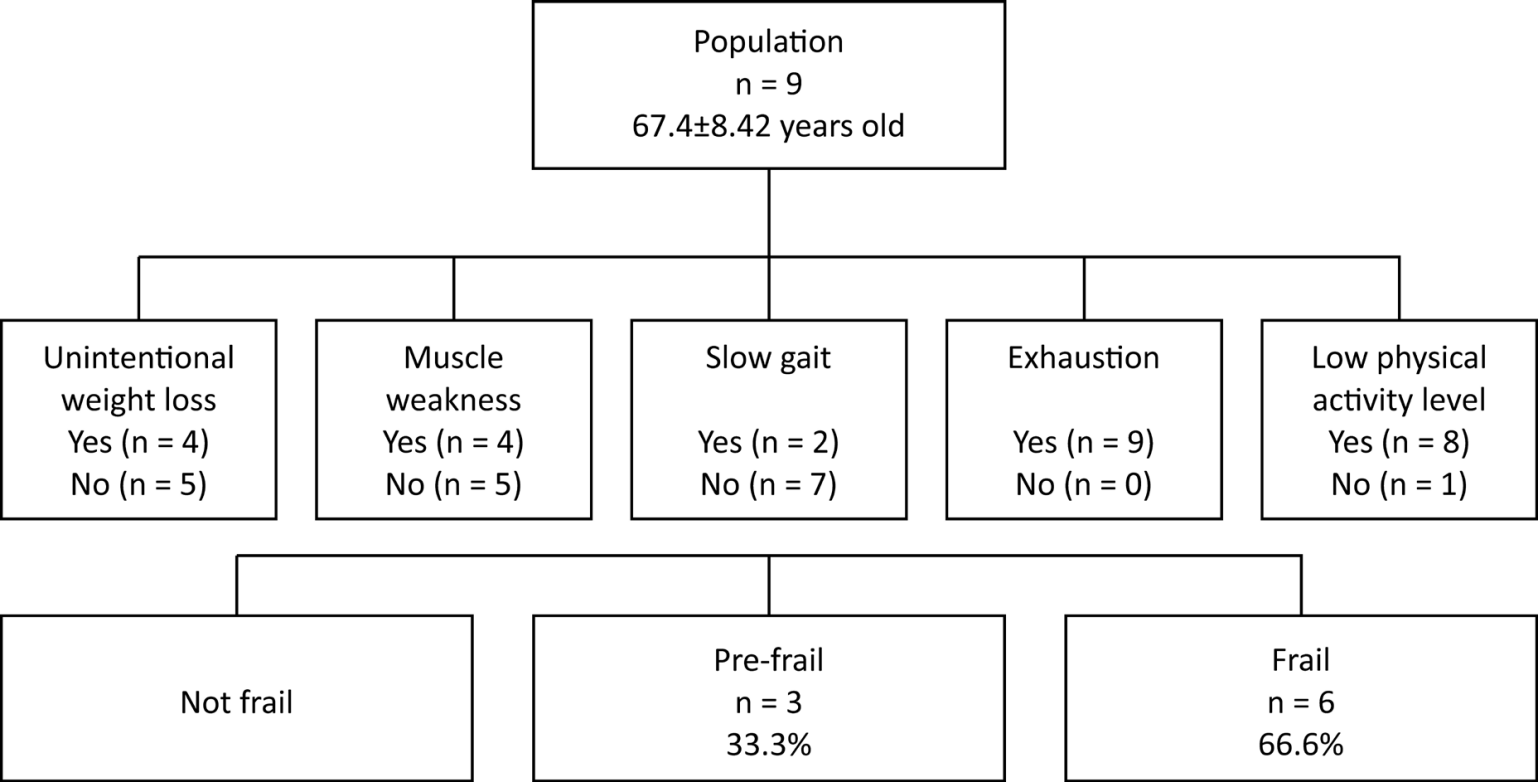
Frailty phenotype according to the Fried criteria.

**Figure 3:**
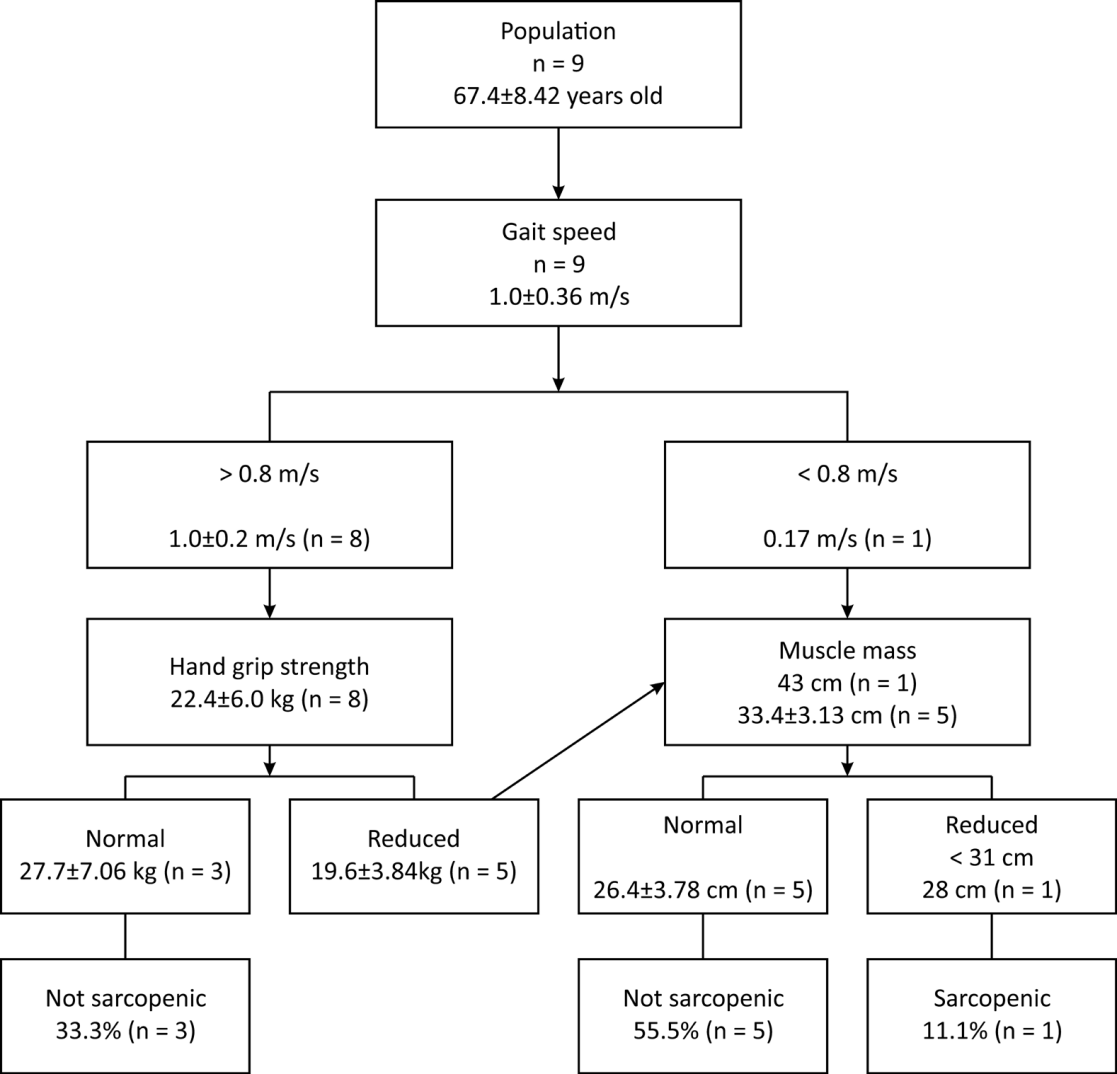
Diagnosis of sarcopenia according to the European Working Group on Sarcopenia in Older People (EWGSOP).

Frailty was more common than sarcopenia and the most common of the Fried criteria used to define the frailty phenotype was exhaustion (100%), followed by low physical activity level (88.8%), muscle weakness (55%), and unintentional weight loss (44.4%). The least frequent criterion was slow GS (22.2%). Sarcopenia screening detected low strength combined with reduced muscle mass in just one patient (11.1%) (Table 1). Taking the entire population, 33.3% were considered pre-frail and 66.6% were diagnosed as frail (Figure 2), whereas sarcopenia was observed in just 11.1% of the sample (Figure 3).

**Table 1:** Diagnostic criteria for sarcopenia and frailty in patients with venous ulcers.

	**Total (n = 9)**
Sarcopenia	
Sarcopenic	11.1% (n = 1)
Not sarcopenic	88.9% (n = 8)
Gait speed (m/s)	1±0.36
Hand grip strength test (kg)	23±5.94
Calf circumference (cm)	36.2±22
Frailty	
Pre-frail	33.3% (n = 3)
Frail	66.6% (n = 6)
Unintentional weight loss	44.4% (n = 4)
Muscle weakness (hand grip strength test)	55.5% (n = 5)
Slowness	22.2% (n = 2)
Exhaustion	100% (n = 9)
Low physical activity	88.8% (n = 8)

## DISCUSSION

Frailty is a biological syndrome that precedes incapacity and is characterized by a high degree of vulnerability to low grade stressors and by clinical manifestations of low functional reserves and low resilience. This high degree of vulnerability is due to changes in multiple physiological systems, primarily inflammation, insulin resistance, coagulation dysfunctions, endothelial dysfunctions, and vascular dysfunctions.^20^

In this study, patients had chronic venous ulcers and, therefore, vascular and endothelial dysfunctions and problems with healing, and it was found that all of the patients were either in a state of pre-frailty (33.3%) or frailty (66.6%). We are not aware of any previous studies of the association between frailty and venous ulcers, but a study conducted with elderly diabetic patients with foot ulcers found frailty and incapacity to perform activities of daily living in this population. Frailty was diagnosed using the Edmonton Frailty Scale and 94% of the patients with ulcers were classified as having mild frailty (42%), moderate frailty (22%), or severe frailty (30%) and diabetic patients without ulcers exhibited mild frailty (24%), moderate frailty (6%), and severe frailty (2%). Patients without diabetes and without ulcers only exhibited mild frailty (10%) and moderate frailty (2%), indicating a strong association between diabetic ulcers and frailty.^21^ The results of the present study show that 33.3% were defined as pre-frail and 66.6% as frail, indicating that chronic venous ulcers are strongly associated with the frailty phenotype.

Exhaustion was the most frequent of the frailty criteria, reported by 100% of the patients. Exhaustion/fatigue was assessed using two questions from the CES-D depression scale, showing that depressive complaints are common in this population. A study^22^ conducted with 60 patients with venous ulcers, 71.6% of whom were elderly women, found that 88.4% had some degree of depression. For many patients, chronic disease, primarily associated with ulcers, involves pain, loss of mobility or functional capacity, and deterioration of quality of life, leading to anxiety and depression. In addition to physical, emotional, and psychological suffering, ulcers also cause disorders of a social nature, since they cause rejection by and isolation from other people.^23^ The association between frailty, depression, and depressive symptomology may be linked to superimposition of other coexisting characteristics onto these health conditions, such as inactivity, weight loss, exhaustion, and low physical activity levels.^24^

Using the criteria for assessment of sarcopenia proposed by the EWGSOP, the results of the present study with elderly patients with chronic venous ulcers only classified one patient (11.1%) as having sarcopenia. The results also showed that the elderly participants in this study had elevated BMI and CC exceeding the cutoff point.

The methods used to assess sarcopenia, which was to use a tape measure to assess CC may not have detected reductions in muscle mass because of lower limb edema, which is characteristic of patients with venous ulcers. It is important to point out that edema is present from CEAP class 3 onwards.^2^ Furthermore, the majority of the patients did have reduced HGS (n = 5), and CC was the criterion that determined whether or not sarcopenia was present. According to the EWGSOP, revised by the 2019 European consensus,^9^ muscle strength became the primary parameter for detection of sarcopenia in relation to muscle mass, because it is recognized that strength is a better predictor of adverse results than mass.

However, the World Health Organization (WHO) considers that CC measurement is sensitive for assessment of muscle mass in the elderly, indicating changes that occur during aging and with reduced physical activity.^6^ In view of the findings of this study, it is suggested that other methods for evaluation of muscle mass be used with this population, such as imaging exams (for example, magnetic resonance or computed tomography). Techniques for assessing the quantity and quality of muscles are primarily available in research centers, rather than clinical environments. Since instruments and methods for assessing muscle quality have been developed and improved over time, it is to be hoped that assessment of muscle mass with greater precision will become more accessible in clinical practice in the future, with greater access to evaluation instruments that employ imaging, thereby offering greater precision and expanding use of muscle mass as a parameter for definition of sarcopenia.^9^

Considering that functional capacity reduces with age and that venous ulcers are most prevalent in this age group, it is necessary to plan strategies to improve patients’ lifestyles, which could increase their autonomy to perform activities of daily living and avoid progression to a clinical status of frailty, thereby minimizing the risk of adverse health events, including falls, hospital admissions, institutionalization, and mortality. Identification and risk stratification of these patients by the healthcare team is important because it can enable better quality treatment and optimized care for fragile patients with ulcers. This requires a systematic and multidimensional approach, focused on functional, psychological, and social elements.^11^

The limitations of this study were the sample size, the heterogeneous sample in terms of the wide age range and the diversity in time since onset of ulcers, absence of measurement of the total area of ulceration and ulcers of different sizes, lack of a control group paired for age and sex, not having used imaging exams for measurement of muscle mass, and not having examined the ankle joint, preventing more extensive conclusions from being drawn.

Considering that sarcopenia and frailty increase the risk of falls and fractures, are detrimental to the ability to perform activities of daily living, and cause mobility disorders, thereby contributing to worse quality of life, loss of independence, or a need for long-term care, and that the great majority of chronic venous ulcers are seen in the elderly population, in terms of practical clinical applicability, simultaneous occurrence of sarcopenia and/or frailty and chronic venous ulcerations contribute to these poor health-related outcomes and it therefore is important that the healthcare professional who treats the chronic ulcers also checks for sarcopenia and frailty and also that researchers investigate this subject, accumulating additional evidence. Furthermore, venous ulcers caused by calf muscle pump insufficiency (muscle weakness) could contribute to sarcopenia occurring earlier and may be identified as another cause of the sarcopenia phenotype in addition to aging. Improving fitness of the calf muscle system could be beneficial for the venous circulatory system, improving the capacity to perform activities of daily living and increasing quality of life.

## CONCLUSIONS

Patients with chronic venous ulcers exhibited the conditions of frailty or pre-frailty and exhaustion, low physical activity level, and muscle weakness were the most frequent of the components that comprise this condition. Sarcopenia was only identified in one patient, which may be a consequence of having assessed muscle mass by the CC method, since patients with venous ulcers frequently have edema of the lower limbs. It is therefore recommended that when screening for sarcopenia in patients with venous ulcers, consideration is given to employing assessment methods that measure the muscular architecture directly. Notwithstanding, these results must be interpreted with caution and confirmed in a study with a larger sample size, with inclusion of sufficient participants with different clinical characteristics. Identification of sarcopenia and frailty in patients with venous ulcers by the healthcare team is important to optimize care and enable better quality treatment for frail and sarcopenic patients with venous ulcers, in order to minimize occurrence of adverse health events.

## References

[1] Sant’Ana SMSC, Bachion MM, Santos QR, Nunes CAB, Malaquias SG, Oliveira BGRB (2012). Úlceras venosas : caracterização clínica e tratamento em usuários
atendidos em rede ambulatorial. Rev Bras Enferm.

[2] Belczak CEQ, Cavalheri G, Godoy JMP, Caffaro RA, Belczak SQ (2007). Relação entre a mobilidade da articulação talocrural e a úlcera
venosa. J Vasc Bras.

[3] O’Brien AJ, Edwards EH, Finlayson JK, Kerr G (2012). Understanding the relationships between the calf muscle pump, ankle
range of motion and healing for adults with venous leg ulcers: a review of the
literature. Wound Pract Res..

[4] Gould LJ, Fulton AT (2016). Wound healing in older adults. RI Med J.

[5] Ribeiro SML, Kehayias JJ (2014). Sarcopenia and the analysis of body composition. Adv Nutr.

[6] Paula JA, Wamser EL, Gomes ARS, Valderramas SR, Cardoso J, Schieferdecker MEM (2016). Análise de métodos para detectar sarcopenia em idosas independentes da
comunidade. Rev Bras Geriatr Gerontol.

[7] Silva LS, Karnikowski MGO, Osório NBB, Pereira LC, Gomide LB, Matheus JPC (2016). Idosos Quilombolas: prevalência de sarcopenia utilizando o algoritmo
proposto pelo European Working Group on Sarcopenia in Older People. Arq Ciências da Saúde..

[8] Dovjak P (2016). arcopenia in cases of chronic and acute illness: a
mini-review. Z Gerontol Geriatr.

[9] Cruz-Jentoft AJ, Bahat G, Bauer J (2019). Sarcopenia: revised European consensus on definition and
diagnosis. Age Ageing.

[10] Achim V, Bash J, Mowery A (2017). Prognostic indication of sarcopenia for wound complication after total
laryngectomy. JAMA Otolaryngol Head Neck Surg.

[11] Gould LJ, Abadir PM, White-Chu EF, Gould LJ, Abadir PM, White-Chu EF (2017). Age, frailty and impaired wound healing. Principles and practice of geriatric surgery.

[12] Tavares DMS, Almeida EG, Ferreira PCS, Dias FA, Pegorari MS (2014). Status de fragilidade entre idosos com indicativo de depressão segundo
o sexo. J Bras Psiquiatr.

[13] Câmara LC, Bastos CC, Volpe EFT (2012). Exercício resistido em idosos frágeis: uma revisão da
literatura. Fisioter Mov.

[14] American College of Sports Medicine – ACSM (2013). ACSM’s guidelines for exercise testing and prescription.

[15] Cavalcanti LM, Pinto FCM, Oliveira GMD, Lima SVC, Aguiar JLDA, Lins EM (2017). Efficacy of bacterial cellulose membrane for the treatment of lower
limbs chronic varicose ulcers: a randomized and controlled trial. Rev Col Bras Cir.

[16] Cruz-Jentoft AJ, Baeyens JP, Bauer JM (2010). Sarcopenia: European consensus on definition and diagnosis Report of
the European Working Group on Sarcopenia in Older People. Age Ageing.

[17] Fried LP, Tangen CM, Walston J (2001). Frailty in older adults: evidence for a phenotype. J Gerontol A Biol Sci Med Sci.

[18] Rolland Y, Lauwers-Cances VÃ, Cournot M (2003). Sarcopenia, calf circumference, and physical function of elderly
women: a cross-sectional study. J Am Geriatr Soc.

[19] Souza AC, Magalhaes LDC, Teixeira-Salmela LF (2006). Adaptação transcultural e análise das propriedades psicométricas da
versão brasileira do *Perfil de Atividade Humana*. Cad Saude Publica.

[20] Angulo J, El Assar M, Rodríguez-Mañas L (2016). Frailty and sarcopenia as the basis for the phenotypic manifestation
of chronic diseases in older adults. Mol Aspects Med.

[21] Bôas NCRV, Salomé GM, Ferreira LM (2018). Frailty syndrome and functional disability among older adults with and
without diabetes and foot ulcers. J Wound Care.

[22] Salomé GM, Blanes L, Ferreira LM (2012). Avaliação de sintomas depressivos em pessoas com úlcera
venosa. Rev Bras Cir Plást.

[23] Aguiar ACSA, Sadigursky D, Martins LA, Menezes TM, Santos AL, Reis LA (2016). Repercussões sociais vivenciadas pela pessoa idosa com úlcera
venosa. Rev Gaúcha Enferm.

[24] Vieira RA, Guerra RO, Giacomin KC (2013). Prevalência de fragilidade e fatores associados em idosos comunitários
de Belo Horizonte, Minas Gerais, Brasil: dados do estudo FIBRA. Cad Saude Publica.

