# Supplementary material for: Screening for sarcopenia and frailty in patients with chronic ulcers: a cross-sectional study
Source: J Vasc Bras. 2020 Jul 31;19:e20190054. doi: 10.1590/1677-5449.190054 (PMC8202172; doi:10.1590/1677-5449.190054)
Supplement: Translation

# Translation: Triagem da sarcopenia e fragilidade em pacientes com úlceras venosas crônicas: um estudo transversal

**Authors:** Tamiris Gomes, Kelly Cristina Blaszkowski Trombini, Marcos Vinicíus Soares Martins, Hilana Rickli Fiuza Martins

**Affiliations:** 1 Faculdade Guairacá, Guarapuava, PR, Brasil.; 2 Universidade Estadual do Centro-Oeste – UNICENTRO, Guarapuava, PR, Brasil.

**Keywords:** úlcera varicosa, sarcopenia, fragilidade

## Abstract

**Contexto:**

Pacientes com úlceras venosas reportam múltiplas comorbidades e são mais propensos a ser fisicamente inativos. A sarcopenia e a fragilidade aumentam a vulnerabilidade de um indivíduo para maior dependência e/ou morte.

**Objetivos:**

Verificar presença da sarcopenia e fragilidade em pacientes portadores de úlceras venosas crônicas.

**Métodos:**

Estudo observacional e transversal, realizado com 9 pacientes com idade média de 67,4 ± 8,42 anos e portadores de úlcera venosa nos membros inferiores classificadas no Consenso Internacional de Doenças Venosas Crônicas (CEAP) em estágio 6. Para identificação e classificação da sarcopenia, foi avaliada a força (dinamometria manual), a velocidade da marcha (teste de caminhada de 10 metros) e a massa muscular (circunferência da panturrilha). Para triagem de fragilidade, foram utilizados os critérios de Fried: perda de peso não intencional, fadiga, redução da força e da velocidade da caminhada e baixa atividade física.

**Resultados:**

O fenótipo de fragilidade foi mais frequente (n = 9; 100%) em relação à sarcopenia (n = 1; 11,1%). Entre os critérios de Fried, os mais frequentes foram a exaustão (n = 9; 100%), seguida pela baixa atividade física (n = 8; 88,8%) e fraqueza muscular (n = 5; 55%). Por fim, o critério menos frequente foi a diminuição da velocidade da marcha (n = 2; 22,2%). No diagnóstico de sarcopenia, foi observada redução da força associada à redução da massa muscular (n = 1; 11,1%).

**Conclusões:**

Pacientes com úlceras venosas crônicas apresentam condição de fragilidade ou pré-fragilidade, enquanto a condição de sarcopenia foi pouco frequente.

## INTRODUÇÃO

As feridas crônicas de causa venosa são as mais frequentes, atingindo índices de até 80% do total de feridas que afetam os membros inferiores e constituem um sério problema de saúde pública em função do grande número de indivíduos acometidos^1^^,^^2^. Úlceras venosas em membros inferiores afetam 1-3% da população com mais de 60 anos e a incidência aumenta com a idade. Por sua vez, os pacientes idosos com úlceras venosas reportam múltiplas comorbidades e estão mais propensos a ser fisicamente inativos^3^.

O desenvolvimento de feridas crônicas é multifatorial e depende de fatores intrínsecos e extrínsecos. Entre os fatores intrínsecos que podem ter um efeito importante na cicatrização de feridas, estão as alterações decorrentes do processo de envelhecimento, como mudanças na composição corporal, desequilíbrio energético, desequilíbrio homeostático e neurodegeneração^4^.

O músculo esquelético pode ser considerado o principal componente proteico do corpo e é capaz de estimular a produção de anticorpos, a cicatrização de feridas e a produção de glóbulos brancos durante doenças agudas ou crônicas. Com a redução da massa muscular com o processo de envelhecimento, denominado sarcopenia, há menor disposição de proteína para prover e manter a funcionalidade e funções fisiológicas^5^. A associação da redução de massa e força muscular resulta em maior risco de quedas, hospitalizações, dependência, institucionalizações, piora da qualidade de vida e mortalidade, repercutindo também nos aspectos sociais e econômicos^6^.

A sarcopenia está associada com o risco de fragilidade, risco de quedas, prejuízos na mobilidade, descontrole glicêmico e metabólico, redução da taxa metabólica basal e capacidade funcional^7^^,^^8^. A sarcopenia aumenta o risco de fraturas, prejudica a capacidade de realizar atividades da vida diária e está associada com doença cardíaca, doença respiratória e comprometimento cognitivo^9^. A investigação da relação entre a sarcopenia e a morbidade cirúrgica em pacientes de cirurgia geral sugere que ela é um fator importante na cicatrização de feridas e complicações^10^.

A fragilidade é um estado clínico de fraqueza e suscetibilidade ao estresse fisiológico decorrente da baixa reserva fisiológica nos sistemas neuromuscular metabólico e imunológico^11^. É uma síndrome clínica de múltiplas causas e é caracterizada por força muscular diminuída e redução da resistência física e função fisiológica, que aumenta a vulnerabilidade de um indivíduo para desenvolvimento de maior dependência e/ou morte^12^. Os aspectos da fragilidade incluem redução da mobilidade, anormalidade na marcha, fraqueza muscular, tolerância reduzida ao exercício, equilíbrio instável, má nutrição e sarcopenia^13^.

Tendo em vista que sarcopenia e fragilidade estão fortemente associadas com efeitos adversos à saúde e interferem na cicatrização de feridas e que as feridas crônicas estão associadas à idade, a comorbidades e à inatividade física, tornando os portadores uma população predisposta à sarcopenia e fragilidade, torna-se necessária a investigação da prevalência dessas condições nessa população, pois, até onde sabemos, não existem estudos prévios que tenham avaliado a relação da sarcopenia e fragilidade em portadores de úlceras venosas. Dessa forma, o presente estudo tem como objetivo investigar a ocorrência de sarcopenia e fragilidade em pacientes portadores de úlceras venosas crônicas.

## MÉTODOS

Este estudo observacional de corte transversal foi realizado com objetivo de avaliar a frequência de sarcopenia e fragilidade em pacientes com úlceras venosas crônicas em membros inferiores. O estudo foi aprovado pelo comitê de ética da Universidade Estadual do Centro-Oeste (UNICENTRO), Guarapuava, PR, Brasil, sob parecer número 2.810.567-2018, e foi realizado nas Clínicas Integradas da Faculdade Guairacá, Guarapuava, PR, Brasil. A amostragem foi não probabilística e o recrutamento da amostra se deu por conveniência por meio de convite verbal aos pacientes do ambulatório de feridas crônicas da mesma instituição em que o estudo foi realizado e por panfletos que foram distribuídos em postos de saúde, clínicas médicas e serviços de saúde. Dezenove pacientes foram contatados ou entraram em contato com a equipe da pesquisa; no entanto, apenas 9 pacientes foram incluídos. A divulgação da pesquisa iniciou no mês de agosto de 2018, após a aprovação do comitê de ética em pesquisa, e o recrutamento da amostra e coleta de dados ocorreram simultaneamente, nos meses de setembro a novembro de 2018.

A pessoa, para ser definida como idosa, deve possuir idade cronológica ≥ 65 anos em países desenvolvidos ou aqueles que possuem de 50 a 64 anos de idade e apresentam condições clínicas ou limitações físicas que afetam a sua locomoção ou a realização das atividades da vida diária, devido ao condicionamento físico ou condições fisiológicas afetadas^14^. Dessa forma, foram incluídos pacientes com idade superior a 50 anos, portadores de úlcera venosa crônica classificada no Consenso Internacional de Doenças Venosas Crônicas (CEAP) 6 (úlcera aberta e ativa)^15^ e que possuíssem diagnóstico médico da condição. Um paciente não possuía o diagnóstico e somente foi inserido no estudo após consulta com um médico cirurgião vascular, que confirmou a condição.

Os critérios de exclusão foram: idade inferior a 50 anos, úlceras em membros inferiores de causa arterial, queimaduras, diabetes e úlceras de pressão. Não participaram do estudo indivíduos que tivessem consumido substâncias que pudessem interferir na marcha, bem como aqueles acometidos por doença metabólica ou endócrina que afetassem o sistema musculoesquelético. Outros fatores de exclusão foram existência de prótese unilateral ou bilateral de quadril, autorrelatos de anormalidades cardíacas e respiratórias, autorrelato de quadro doloroso agudo nos membros inferiores ou superiores, amputação de membros superior e/ou inferior, acidente vascular encefálico (AVE), Parkinson, caquexia cancerosa, doença renal crônica, Alzheimer ou doença psiquiátrica, artrite grave ou doença inflamatória e anorexia por medicação, falta de transporte para que pudessem ir até o local das avaliações ou aqueles que não concordassem em participar da pesquisa.

A sarcopenia foi diagnosticada pelo algoritmo proposto pelo *European Working Group on sarcopenia in Older People* (EWGSOP)^16^ e a fragilidade, pelo fenótipo de fragilidade de Fried et al.^17^. Para a triagem de sarcopenia, foram utilizados os valores da velocidade da marcha (VM), força de preensão manual (FPM) e circunferência da panturrilha (CP), de acordo com o proposto pelo Consenso Europeu. A presença de sarcopenia foi definida quando o paciente apresentou diminuição de massa muscular esquelética associada à redução de força e/ou desempenho físico. Para estadiamento da sarcopenia, foram consideradas pré-sarcopenia (redução de massa muscular), sarcopenia (diminuição de massa muscular associada à redução de força e/ou desempenho físico) e sarcopenia grave (redução de massa muscular esquelética, força muscular e desempenho físico).

A Síndrome da Fragilidade foi identificada de acordo com os cinco critérios propostos por Fried et al.^17^: perda de peso não intencional; exaustão avaliada por autorrelato de fadiga; diminuição da FPM; baixo nível de atividade física; diminuição da velocidade de caminhada. Os pacientes foram classificados em frágeis quando três ou mais critérios estavam presentes, pré-frágeis quando um ou dois estavam presentes e não frágeis quando nenhum critério estava presente.

A força de preensão (kg) foi avaliada com o dinamômetro digital (Camry, Modelo EH101, China). Os participantes se sentaram confortavelmente, com cotovelos fletidos em um ângulo de 90° junto ao corpo. Foram realizadas três tentativas com 1 minuto de descanso entre elas e o valor considerado para a análise foi a média das três. A redução da força muscular foi definida de acordo com o sexo e índice de massa corporal [IMC = massa corporal (kg)/estatura^2^ (m)]. Os pontos de corte para mulheres foram: ≤ 17 kg para IMC ≤ 23kg/m^2^; ≤ 17,3 kg para IMC entre 23,1-26 kg/m^2^; ≤ 18 kg para IMC entre 26,1-29 kg/m^2^ e ≤ 21 kg para IMC > 29. Os pontos de corte para homens foram ≤ 29 kg para IMC ≤ 24 kg/m^2^; ≤ 30 kg para IMC entre 24,1-26 kg/m^2^; ≤ 30 kg para IMC entre 26,1-28 kg/m^2^ e ≤ 32 kg para IMC > 28^16^.

A VM foi avaliada pelo teste de caminhada de 10 metros, em que os participantes caminharam, em linha reta, uma distância de 10 metros. Os dois primeiros e os dois últimos metros foram excluídos da análise, respeitando as fases de aceleração e desaceleração e, assim, o tempo para percorrer o percurso de 6 metros foi registrado. A distância foi dividida pelo tempo que o participante levou para concluir o percurso para fornecer a medida da velocidade em m/s. O teste foi realizado três vezes e foi considerada como VM reduzida o valor inferior a ≤ 0,8 m/s^6^^,^^16^.

A CP foi aferida com o auxílio de uma fita métrica inelástica contornando a maior curvatura da panturrilha. O participante se sentou em uma cadeira, com o quadril e joelho em uma posição de 90°. Foram considerados valores menores que 31 cm como indicativos de depleção de massa muscular^18^.

A perda de peso não intencional foi avaliada por meio da pergunta direta ao participante sobre a ocorrência de perda de 4,5 kg ou mais no último ano ou pelo menos 5% do peso corporal, e, no caso de resposta afirmativa, foi considerada como um critério de fragilidade. Para tanto, também foi realizada a avaliação da massa corporal utilizando-se uma balança digital (Filizola, Brasil), com precisão de 0,1 kg. A estatura também foi avaliada com um estadiômetro (Cardiomed, Brasil). Após, foi calculado o IMC.

A exaustão/fadiga foi obtida por duas questões da escala de depressão do Centro de Estudos Epidemiológicos (*Center for Epidemiologic Studies-Depression*, CES-D). Os participantes foram questionados, perguntando-se: “Sentiu que precisou fazer esforços para dar conta das atividades habituais?” e “Não conseguiu levar adiante suas coisas?”. As respostas foram avaliadas pela escala Likert (nunca ou raramente = 1, às vezes = 2, frequentemente = 3, sempre = 4). Se o paciente respondeu na maioria das vezes e/ou sempre para uma das duas questões, a fadiga foi considerada como critério de fragilidade.

O nível de atividade física foi avaliado através do Perfil de Atividade Humana (PAH), questionário adaptado e validado para a população idosa brasileira. Esse questionário é constituído por 94 itens, que variam desde atividades rotineiras de nível funcional baixo (levantar e se sentar em cadeira ou cama sem ajuda) até atividades de nível funcional mais alto (correr 4,8 quilômetros em menos de 30 minutos). Tais atividades estão baseadas no custo energético: as de menor numeração demandam menor gasto energético, as de numeração mais alta, maior gasto energético. O idoso foi considerado ativo quando escore ajustado de atividades (EAA) > 74; moderadamente ativo quando 53 > EAA < 74 e inativo quando EAA< 53^19^.

A velocidade de caminhada foi avaliada pela VM cronometrada para percorrer 4,6 metros em passo usual. Receberam 1 ponto homens com ≤ 173 cm de estatura que levaram ≥ 7 segundos ou homens com ≥ 173 cm de estatura que levaram ≥ 6 segundos para percorrer a distância de 4,6 metros. Receberam 1 ponto mulheres com ≤ 159 cm que levaram ≥ 7 segundos ou mulheres com ≥ 159 cm que levaram ≥ 6 segundos para percorrer a distância de 4,6 metros^17^.

Para a análise dos resultados, foi utilizada estatística descritiva. Para a análise descritiva das características da população, foram calculadas as frequências absolutas e relativas, média e desvio padrão. A análise estatística foi realizada por meio do pacote estatístico SPSS versão 23.

## RESULTADOS

Nove pacientes com úlcera venosa nos membros inferiores participaram do estudo, sendo 66,7% (n = 6) do sexo feminino e 33,3% (n = 3) do sexo masculino, com média de idade de 67,44±8,42 anos, massa corporal de 71,2±28,8 kg e estatura de 1,63±0,11 metros (Figura 1). Dos nove participantes, 66,7% (n = 6) apresentavam ferida unilateral e 33,3% (n = 3) apresentavam ferida bilateral. O tempo médio de ferida ativa foi de 117,8±160,33 meses e o número de feridas ativas foi de 2,77±3,27. Em relação às doenças associadas, 66% (n = 6) dos pacientes apresentavam hipertensão arterial e 22,2% (n = 2), doença cardiovascular.

**Figura 1:** Fluxograma do estudo.

A fragilidade foi mais frequente em relação à sarcopenia e, entre os critérios de Fried para definição do fenótipo de fragilidade, os mais frequentes foram exaustão (100%), seguida pela baixa atividade física (88,8%), fraqueza muscular (55%) e perda de peso não intencional (44,4%). O critério menos frequente foi a diminuição da VM (22,2%). No diagnóstico de sarcopenia, foi observada redução da força associada à redução da massa muscular em apenas um paciente (11,1%) (Tabela 1). Da população total, 33,3% foram considerados como pré-frágeis e 66,6%, como frágeis (Figura 2), enquanto a sarcopenia foi observada em apenas 11,1% da amostra (Figura 3).

**Tabela 1:** Critérios diagnósticos da sarcopenia e fragilidade em pacientes com úlceras venosas.

	**Total (n = 9)**
Sarcopenia	
Sarcopênico	11,1% (n = 1)
Não sarcopênico	88,9% (n = 8)
Velocidade da marcha (m/s)	1±0,36
Força de preensão manual (kg)	23±5,94
Circunferência da panturrilha (cm)	36,2±22
Fragilidade	
Pré-frágil	33,3% (n = 3)
Frágil	66,6% (n = 6)
Perda de peso não intencional	44,4% (n = 4)
Fraqueza muscular (força de preensão manual)	55,5% (n = 5)
Lentidão	22,2% (n = 2)
Exaustão	100% (n = 9)
Baixa atividade física	88,8% (n = 8)

**Figura 2:** Fenótipo de fragilidade, de acordo com os critérios de Fried.

**Figura 3:** Diagnóstico de sarcopenia de acordo com o *European Working Group on Sarcopenia in Older People* (EWGSOP).

## DISCUSSÃO

A fragilidade é uma síndrome biológica que precede a incapacidade e é caracterizada por uma alta vulnerabilidade a estressores de baixa potência, bem como pela manifestação clínica de diminuição da reserva funcional e resiliência. Essa alta vulnerabilidade é devida a mudanças em vários sistemas fisiológicos, principalmente inflamação, resistência à insulina, alterações na coagulação e disfunção endotelial e vascular^20^.

Neste estudo com pacientes com úlceras venosas crônicas e, portanto, apresentando disfunção vascular, endotelial e alteração na cicatrização, foi encontrado que todos os pacientes apresentavam condição de pré-fragilidade (33,3%) ou fragilidade (66,6%). Estudos sobre a associação da fragilidade e úlceras venosas são desconhecidos, mas um estudo realizado com pacientes idosos diabéticos com úlceras em pé identificou fragilidade e incapacidade para as atividades da vida diária nessa população. O diagnóstico da fragilidade foi realizado com a *Edmonton Frailty Scale* (EFS), e 94% dos pacientes com úlceras foram classificados como fragilidade leve (42%), fragilidade moderada (22%) e fragilidade grave (30%), enquanto os pacientes diabéticos sem úlceras apresentaram fragilidade leve (24%), fragilidade moderada (6%) e fragilidade grave (2%). Já os pacientes não diabéticos e sem feridas apresentaram fragilidade leve (10%) e fragilidade moderada (2%) apenas, indicando a forte associação de úlceras diabéticas com fragilidade^21^. Os resultados do presente estudo mostraram que 33,3% foram considerados como pré-frágeis e 66,6% como frágeis, indicando que úlceras venosas crônicas estão fortemente associadas com o fenótipo de fragilidade.

Entre os critérios da fragilidade, a exaustão foi o mais frequente, sendo relatada por 100% dos pacientes. A exaustão/fadiga foi obtida por duas questões da escala de depressão do CES-D, mostrando que queixas depressivas estão comumente presentes nessa população. Um estudo^22^ realizado com 60 pacientes com úlceras venosas, sendo 71,6% da amostra representada por pessoas idosas, mostrou que 88,4% apresentava algum nível de depressão. Para muitos pacientes, a doença crônica, associada, principalmente, a ferida, significa dor, perda de mobilidade ou da capacidade funcional e piora da qualidade de vida, resultando em ansiedade e depressão. Além do sofrimento físico, emocional e psicológico, a úlcera também causa transtornos de cunho social, uma vez que causa rejeição e afastamento de outras pessoas^23^. A associação entre fragilidade, depressão e sintomatologia depressiva poderia estar vinculada à sobreposição de características coexistentes em tais condições de saúde, como a inatividade, a perda de peso, a exaustão e o nível reduzido de atividade física^24^.

Seguindo os critérios propostos pelo Consenso Europeu para avaliar sarcopenia, os resultados do presente estudo realizado com idosos com feridas venosas crônicas mostrou que apenas um paciente (11,1%) foi classificado como sarcopênico. Os resultados também mostraram que os idosos participantes desse estudo apresentaram um elevado IMC e uma CP acima do ponto de corte.

Dos métodos utilizados para avaliação da sarcopenia, o uso da fita métrica para avaliação da CP pode não ter identificado redução da massa muscular pela ocorrência de edema em membros inferiores, característica dos pacientes com úlceras venosas. É importante ressaltar que, a partir de CEAP 3, existe edema^2^. Ainda, a maior parte dos pacientes apresentaram redução da FPM (n = 5), e o critério que definiu a não ocorrência de sarcopenia foi a CP. De acordo com a EWGSOP, revisada pelo consenso europeu em 2019^9^, a força muscular passa a se apresentar como parâmetro primário para a detecção da sarcopenia em relação à massa muscular, pois é reconhecido que a força é melhor do que a massa na previsão de resultados adversos.

No entanto, a Organização Mundial da Saúde (OMS) considera a medida da CP sensível para avaliar a massa muscular nos idosos, a qual indica alterações que ocorrem com o envelhecimento e com a redução de atividade física^6^. Diante dos achados deste estudo, sugere-se que outros métodos de avaliação da massa muscular sejam utilizados nessa população, como exames de imagem (por exemplo, ressonância magnética ou tomografia computadorizada). Técnicas para avaliar a quantidade e qualidade muscular estão disponíveis principalmente em centros de pesquisa, mas não em todos os ambientes clínicos. Como instrumentos e métodos para avaliar a qualidade muscular têm sido desenvolvidos e aperfeiçoados ao longo do tempo, espera-se que no futuro a avaliação da massa muscular com maior precisão se torne mais acessível na pratica clínica, com maior acesso à instrumentos de avaliação que façam uso de imagens permitindo, assim, maior precisão e maior utilização do parâmetro massa muscular para definição da sarcopenia^9^.

Tendo em vista que a capacidade funcional diminui com a idade e que úlceras venosas são mais prevalentes nessa faixa etária, é necessário planejar estratégias para melhorar o estilo de vida dos pacientes, o que pode aumentar a autonomia em suas atividades da vida diária e evitar o quadro clínico de fragilidade, minimizando, assim, o risco para resultados adversos à saúde, incluindo quedas, hospitalização, institucionalização e mortalidade. A identificação e estratificação de risco desses pacientes pela equipe de saúde têm sua importância no sentido de proporcionar melhor qualidade no tratamento e otimizar o atendimento para o paciente frágil portador de feridas, o que requer uma sistemática abordagem multidimensional, com foco nos componentes funcionais, psicológicos e sociais^11^.

As limitações do estudo foram o tamanho da amostra, a amostra heterogênea devido à faixa etária extensa e a dispersão relativa no tempo de ferida, ausência de mensuração da área total ulcerada e úlceras de tamanhos diferentes, ausência de grupo controle pareado por sexo e idade, não utilização de exames de imagem para mensuração da massa muscular e ausência da avaliação da articulação tibiotársica, o que acarreta a impossibilidade de conclusões abrangentes.

Tendo em vista que a sarcopenia e a fragilidade aumentam o risco de quedas e fraturas, prejudicam a capacidade de realizar atividades de vida diária, levam a distúrbios de mobilidade, contribuindo, assim, para a diminuição da qualidade de vida, a perda de independência ou a necessidade de cuidados de longo prazo, e que as úlceras venosas crônicas acometem em sua grande maioria a população idosa, em termos de aplicabilidade clínica prática, a ocorrência simultânea da sarcopenia e/ou fragilidade e ulcerações venosas crônicas contribuem para esses desfechos ruins relacionados à saúde, sendo importante ao profissional de saúde que trata da condição de úlcera crônica verificar também a ocorrência de sarcopenia e fragilidade, assim como aos pesquisadores investigarem essa temática, proporcionando maiores evidências. Ainda, as úlceras venosas caracterizadas pela insuficiência da bomba de panturrilha (fraqueza muscular) podem favorecer a ocorrência mais cedo da sarcopenia, podendo ser identificada como uma causa do fenótipo da sarcopenia além do envelhecimento. Melhorias no condicionamento do sistema muscular da panturrilha podem ser benéficas para o sistema circulatório venoso, melhorando a capacidade de realizar atividades de vida diária e proporcionando um aumento na qualidade de vida.

## CONCLUSÃO

Pacientes com úlceras venosas crônicas apresentaram condição de fragilidade ou pré-fragilidade e, entre os componentes que integram essa condição, a exaustão, a baixa atividade física e a fraqueza muscular foram os mais frequentes. A condição de sarcopenia foi identificada em apenas um paciente, o que pode ter sido consequente à avaliação da massa muscular pelo método da CP, pois pacientes com úlceras venosas frequentemente apresentam edema em membros inferiores. Dessa forma, recomenda-se que, para triagem da sarcopenia em pacientes com úlceras venosas, sejam considerados métodos de avaliação que mensurem a arquitetura muscular de forma direta. No entanto, esses resultados precisam ser interpretados com cautela e confirmados por um estudo com maior tamanho amostral, com a inclusão suficiente de participantes com diferentes características clínicas. A identificação de sarcopenia e fragilidade em pacientes com úlceras venosas pela equipe de saúde tem importância no sentido de otimizar o atendimento e proporcionar melhor qualidade no tratamento para pacientes frágeis e sarcopênicos portadores de úlceras venosas, a fim de minimizar a ocorrência de eventos adversos à saúde.
